# Contribution of the *srtBCD* pilus cluster genes *sbp1* and *sbp3* to the virulence of *Streptococcus suis* serotype 2 sequence type 1

**DOI:** 10.1186/s13567-026-01891-y

**Published:** 2026-11-13

**Authors:** Parichart Boueroy, Nattamol Phetburom, Peechanika Chopjitt, Rujirat Hatrongjit, Yukihiro Akeda, Nahuel Fittipaldi, Mariela Segura, Marcelo Gottschalk, Kazunori Oishi, Anusak Kerdsin

**Affiliations:** 1 Department of Health Science, Faculty of Public Health, Kasetsart University, Chalermphrakiat Sakon Nakhon Province Campus, Sakon Nakhon, Thailand; 2 Special Research Unit for Tropical Zoonoses and Antimicrobial Resistance Surveillance (TZARS), Kasetsart University, Bangkok, Thailand; 3 Faculty of Veterinary Science, Chulalongkorn University, Bangkok, Thailand; 4 Faculty of Science and Engineering, Kasetsart University, Chalermphrakiat Sakon Nakhon Province Campus, Sakon Nakhon, Thailand; 5 Department of Bacteriology I, National Institute of Infectious Diseases, Tokyo, Japan; 6 Research Group On Infectious Diseases in Production Animals (GREMIP), and Swine and Poultry Infectious Diseases Research Centre (CRIPA), Faculty of Veterinary Medicine, University of Montreal, Quebec, QC Canada; 7 Toyama Institute of Health, Toyama, Japan

**Keywords:** *Streptococcus suis*, sequence type, serotype, *srtBCD*, *sbp1*, *sbp3*

## Abstract

*Streptococcus suis* is an important zoonotic bacterial pathogen that causes substantial economic losses to the porcine industry and serious invasive infection in humans. Many virulence factors have been characterized as being involved in *S. suis* virulence and pathogenesis. Among these, the *srtBCD* pilus cluster gene, which encodes three type C sortases (*srtB, srtC*, and *srtD*) and four pilin subunit proteins, *sbp1-sbp4*, is associated with disease-causing *S. suis* isolates. In this study, we constructed mutant strains devoid of *sbp1* and *sbp3* in a virulent *S. suis* serotype 2 sequence type 1 strain, and evaluated the impact on virulence using both in vitro and in vivo models of infection. This study identifies *sbp1* and *sbp3* contribute to host–pathogen interactions. Inactivation of either gene reduced the bacterium’s ability to damage epithelial cells, attach to host cells, and invade them, demonstrating their role in the early stages of infection. In addition, inactivation of *sbp1* or *sbp3* decreased the expression of the sortase-associated gene cluster (*SSU1889, SSU1886, srtB, srtC, srtD*) and several virulence-related genes, including the adhesin gene *ssfsb*, *eno* and the cytolysin gene *sly*, suggesting that these proteins influence a broader network. Among the two proteins, *sbp1* appeared to have a greater impact on virulence, as the *Δsbp1* mutant showed stronger reductions in virulence gene expression and caused lower mortality in mice, although statistical does not significant, suggesting that *sbp1* plays a more prominent role than *sbp3* in pathogenicity.

**Supplementary Information:**

The online version contains supplementary material available at https://doi.org/10.1186/s13567-026-01891-y.

## Introduction

*Streptococcus suis* is an important zoonotic bacterial pathogen that causes substantial economic losses to the porcine industry and may cause serious invasive infections in humans. A plethora of different virulence factors have been proposed to contribute to *S. suis* virulence and pathogenesis [1, 2]. The candidate that has been best studied is the bacterial capsule, which is essential for resistance to phagocytosis [3]. The capsule is also the basis of *S. suis* serotyping, with 29 officially  recognized serotypes currently described [4]. Of them, serotype 2 is the most frequently isolated from diseased pigs and human patients [5, 6], and many studies have characterized virulent factors that are involved in virulence and pathogenesis with this serotype [1–3].

A recent genomic meta-analysis study described a total of 124 factors potentially involved in *S. suis* virulence [2]. A subset of 26 factors was more prevalent in isolates recovered from human patients than in isolates recovered from pigs, and were therefore proposed to represent putative zoonotic virulence factors (PZVFs) [2]. Included among the 26 PZVFs were genes *sbp1* and *sbp2*, both members of a genetic locus known as the *srtBCD* pilus cluster [2]. The *srtBCD* pilus cluster was first described in 2009 [7], and appears to be uniquely found in *S. suis* strains belonging to the clonal complex (CC) 1, a recognized highly virulent group of genetic lineages whose founder is sequence type (ST) 1, and which is responsible for infections in swine and humans worldwide [7, 8]. The *srtBCD* pilus cluster is composed of *srtB*, *srtC*, and *srtD* (encoding class C sortase genes) and *sbp1*, *sbp2’*, *sbp2″*, *sbp*3, and *sbp4* (putative prepilin genes) [7].

Previous studies have shown that the *srtBCD* pilus cluster is a valuable marker permitting the distinction between virulent and non-virulent strains, with *sbp2'* being a potential marker of virulent isolates [9, 10]. Inactivation of the *srtBCD* pilus cluster impairs interactions of *S. suis* serotype 2 ST7 strain with human laryngeal epithelial cells, suggesting a role in pathogen-host interaction [11]. *sbp2′* represents the 5′ portion of a pseudogene that, if uninterrupted, would together with *sbp2″,* encode a pilin subunit; however, available data suggest that a truncated Sbp2′ is expressed and may contribute to the pathogenesis of ST1 *S. suis* serotype 2 [12]. In addition, *sbp1* was identified as an adhesion-associated factor that does not impair the virulence of a *S. suis* serotype 2 ST7 strain [13].

In a previous investigation, we demonstrated that the *srtBCD* pilus cluster was present in a Thai serotype 2 ST1 strain 26154, but absent from ST104 strains, which are epidemiologically associated with Thailand yet genetically distinct from the highly virulent CC1 lineage [14]. Although the association of the *srtBCD* cluster with CC1 virulent lineages is well established, the functional contribution of its individual pilus-associated components remains poorly defined. To better understand the role(s) of *sbp1* and *sbp3* in the pathogenesis of ST1 *S. suis* serotype 2, we constructed deletion mutants of *sbp1* and *sbp3* in the serotype 2 Thai strain 26154. We assessed their roles in biofilm formation, virulence-associated gene transcription, cytotoxicity, adhesion, and invasion of lung epithelial cells, as well as their contribution to virulence in a murine infection model.

## Materials and methods

### Bacterial strains, cell line, and growth conditions

*S. suis* strain 26154 (ST1, serotype 2, henceforth the wild-type strain) was cultured in Todd-Hewitt broth (BD Bacto™, Becton, Dickinson and Company, USA) at 37 °C with 5% CO_2_. Preparation of *S. suis* competent cells was performed as described elsewhere [15, 16]. *Escherichia coli* DH5α strain was cultured in Luria–Bertani broth (HiMedia Laboratories, Mumbai, India) and used for propagation of plasmid vectors and constructs. For mutagenesis, antibiotics (Sigma-Aldrich, St. Louis, MO, USA) were added to the media at following concentration: chloramphenicol at 4 μg/mL and spectinomycin at 100 μg/mL for *S. suis*. For *E. coli,* spectinomycin at 50 μg/mL. Growth curves of *S. suis* WT and mutant derivative strains were determined by assessment of OD 600_ nm_ every hour for 12 h after inoculation of strains into Todd-Hewitt broth and incubation at 37 °C with 5% CO_2_.

The human lung adenocarcinoma cell line A549 (ATCC, CCL-185, Manassas, VA, USA) were cultured in 25-cm^2^ flasks containing Roswell Park Memorial Institute − 1640 medium (Gibco Life Technologies Corporation, Grand Island, NY, USA), supplemented with 15% fetal bovine serum (Gibco), 100 units/mL penicillin, and 100 µg/mL streptomycin (Gibco). The cells were incubated at 37 °C in a humidified atmosphere containing 5% CO_2_.

### DNA and plasmid preparations, and construction of mutagenesis vectors

DNA was prepared from the WT strain using the DNeasy Blood and Tissue kit (Qiagen, Hilden, Germany) according to the manufacturer’s instructions. Plasmid preparations were carried out with the QIAprep Plasmid Miniprep Kit (Qiagen, Hilden, Germany) following the manufacturer’s instructions. A schematic diagram for the construction of mutagenesis vectors is shown in Additional file 1. Briefly, overlap-extension PCR [17] was used to fuse PCR amplified products of upstream and downstream regions of either *sbp1* or *sbp3* with gene *cat*, conferring chloramphenicol resistance, and amplified from plasmid pSET5s. All primers are described in Table 1. For *sbp1*, the fused construct was digested with *Pst*I and *Bgl*II, then cloned into the thermosensitive suicide vector pSET4s digested with the same enzymes, giving rise to vector pSBP1 (pSET4s::*Δsbp1*). A similar approach was used for *sbp3*, but the fused construct was digested with *Sph*I and *Sma*I, then cloned into the same sites of pSET4s, giving rise to vector pSBP3 (pSET4s::*Δsbp3*). PCR with the primer pair described in Table 1 and DNA sequencing were used to verify both mutagenesis constructs.

**Table 1:** **Primers used in this study**

Primer name	Sequence (5′–3′)	Target gene
sbp1- *Pst*I- NF	GAT CAA CTG CAG ATG AAT GTA GAA TTG ATC CCC GAC	*sbp1*
sbp1-NR	CGT TTG ACA GAC TAG AAT AGA GGG	
sbp1-CF	CTG CCC CTC CTT TCT AGT AAT AAT T	
sbp1- *Bgl*II-CR	CCT GGA AGA TCT GTG CAA CAT TCA CTC TAG CTA ATG GC	
sbp1N-cat-F	GGG ATT TTC CCT CTA TTC TAG TCT GTC AAA CGC CGA TAA TTC GAT GGG TTC CGA GG	
sbp1C-cat-R	GCT TAA TTA TTA CTA GAA AGG AGG GGC AGC ACC GAA CTA GAG CTT GAT G	
sbp3- *Sph*I-NF	CAT ACA CAT GCA TGC CAT GGA CTA AGT GTT TAA TAT TTA TCA G	*sbp3*
sbp3-NR	ACG GTT TGA GTG CAC CGG TAT TGT TGG	
sbp3-CF	TGC ATC AAT TTT CTG CAC TTT AAC TTT A	
sbp3- *Sma*I-CR	AAG TCC CCC GGG AGG AGA AAA TTT TAT GAT TTT CTT CAT CC	
sbp3N-cat-F	AAC TCC AAC AAT ACC GGT GCA CTC AAA CCG TCC GAT AAT TCG ATG GGT TCC GAG G	
sbp3C-cat-R	TTA TAA AGT TAA AGT GCA GAA AAT TGA TGC ACA CCG AAC TAG AGC TTG ATG	
sbp1KO-check-F	CTT GAA ACT CCT GCA CTG ACA	Upstream of *sbp1* and *cat*
CH2	CAC CGA ACT AGA GCT TGA TG	
sbp1KO-check-R	ACA ATC CTC AAG CGA CAG CA	Downstream of *sbp1* and *cat*
CH1	TAA TTC GAT GGG TTC CGA GG	
sbp3KO-check-F	GCT GCG TCC TTA GGG ACT TT	Upstream of *sbp*3 and *cat*
CH2	CAC CGA ACT AGA GCT TGA TG	
sbp3KO-check-R	ACC GAA GCT GTT CCA TCA GG	Downstream of *sbp*3 and *cat*
CH1	TAA TTC GAT GGG TTC CGA GG	
sly-F	GCTTGACTTACGAGCCACAA	*sly*
sly-R	CCGCGCAATACTGATAAGC	
Ssfsb-F	TCC RAT RCT GCT CTG CCA TT	*ssfsb*
Ssfsb-R	TGA TAG TAG AAG TCC AGC ARA CT	
Eno-F	ACT CAC GCG GTA ACC CTA CA	*eno*
Eno-R	CCG ATG ATA GCG TCA GCA A	
srtD-F	TGC CGG TCA CCG TAG TTG GT	*srtD*
srtD-R	TAC GTC AGC GTC CTC CCA CC	
srtC-F	GGC TGG GGT AGT GGA ATA CGC T	*srtC*
srtC-R	GGA AGA CCA CGA TGC GCC GTA	
srtB-F	GGG GGA GCG ATA CCC ATA CG T	*srtB*
srtB-R	GC CCA GGC TTG ACC AGC AA	
SSU1886-F	TGC TCG CGA CAC TC TTA CGA	*SSU1886*
SSU1886-R	ACA GGC GAA GTA GGG AGA GCG	
SSU1889-F	AGC TCA CAA GCT GCC CAT GA	*SSU1889*
SSU1889-R	CCG CAC CG CCG GAA CAT TG	

### Generation of *sbp1* and *sbp3* mutant strains

We followed previously described methods for the generation of mutant strains [15]. Briefly, pSBP1 and pSBP3 were electroporated into the WT strain 26154. Single-crossover integrants were selected by incubation on Todd Hewitt agar containing spectinomycin at 37 °C (non-permissive temperature for replication of pSET4s derivatives in Gram-positive bacteria). These integrants were then serially passaged at 28 °C (the permissive temperature) on sheep-blood agar without spectinomycin to promote plasmid excision and resolution to double-crossover mutants (Additional file 1). Deletion inactivation of *sbp1* or *sbp3* was confirmed by PCR with the primers shown in Table 1 and DNA sequencing.

### Biofilm formation assay

Biofilm formation by the *S. suis* strains WT, *Δsbp1* and *Δsbp3* was assessed as previously described [16]. Briefly, bacterial suspensions were added to 96-well flat-bottom microplate and incubated at 37 ℃ in 5% CO_2_ for 2, 4, 24, 48, and 72 h. After incubation, the supernatant was discarded and the biofilms were washed three times with distilled water. The biofilms were fixed, air-dried, and stained with 200 μL of 0.1% crystal violet at room temperature for 30 min. After staining, the biofilm-bound crystal violet was solubilized with 200 μL of 33% (v/v) glacial acetic acid (RCI Labscan Ltd., Bangkok, Thailand), and the biofilms were quantified by recording the absorbance at 595 nm using a Multiskan SkyHigh full-wavelength microplate reader (A51119500C, ThermoFisher Scientific, Waltham, MA, USA).

### Quantitative real-time polymerase chain reaction (RT-qPCR)

Total RNA was extracted from the mid-log phase of *S. suis* WT, *Δsbp1*, and *Δsbp3* strains by using easy-BLUE™ total RNA extraction kit (iNtRON Biotechnology, Gyeonggi-do, Korea). cDNAs were created by reverse transcription with Maxime™ RT PreMix kit (iNtRON Biotechnology, Gyeonggi-do, Korea) according to the manufacturer protocols. The specific primer of srtBCD cluster genes (*SSU1889, SSU1886, srtB, srtC, srtD*), adhesin genes (*ssfsb, eno*), and suilysin gene *(sly*) is shown in Table 1. Quantitative PCR (qPCR) was conducted to evaluate the transcriptional levels of *these genes* on the QuantStudio™ 1 Real-Time PCR System (Applied biosystem, Singapore) using a SYBR Green qPCR Master Mix kit (qPCRBIO SyGreen Mix Lo-ROX, London, UK). The RT-PCR program involved initial denaturation at 95 °C for 2 min, followed by 40 cycles of 95 °C for 15 s, 55 °C for 15 s and 72 °C for 45 s, and ended with the melting curve step 95 °C for 15 s, 60 °C for 1 min, and 95 °C for 15 s. The 16S rRNA gene was used as the internal control. The mRNA levels were presented as relative fold changes using the 2^− ΔΔ Ct^ method [16].

### Cytotoxicity of *S. suis* to human epithelial cells

We used a previously described cytotoxicity assay, with some modifications [18]. Briefly, A549 cells were seeded at 1 × 10^4^ cells/well on a 96-well tissue culture plate (Corning Incorporated, Corning, NY, USA) and incubated overnight (12 h) at 37 °C and 5% CO_2_. A549 cells were infected with *S. suis* WT, *Δsbp1*, and *Δsbp3* mutant strains, collected during the mid-log phase and prepared at a multiplicity of infection (MOI) of 1 (1 × 10^4^ CFU/mL), 10 (1 × 10^5^ CFU/mL), and 100 (1 × 10^6^ CFU/mL). The interactions were allowed to proceed for 2, 4, or 18 h, and the effect of *S. suis* on these cells was determined using the CCK-8 assay (Dojindo Molecular Technologies, Inc.; Rockville, MD, USA), performed as per the manufacturer’s recommendations.

### Adhesion and invasion activity of the *sbp*-knockout strains

To assess adhesion and invasion we used a previously described assay with some modification [18]. Briefly, A549 cells were seeded at 1 × 10^5^ cells/well on a 6-well tissue culture plate (Corning Incorporated, Corning, NY) containing culture medium and incubated overnight (12 h) at 37 °C and 5% CO_2_ to generate 95% confluent cells. Mid-log phase cultures of *S. suis* (WT, *Δsbp1*, and *Δsbp3*) were centrifuged at 2000 rpm for 5 min, washed twice with 1 × phosphate-buffered saline (PBS), resuspended in RPMI, and co-cultured with A549 cells at MOI 1 (1 × 10^5^ CFU/mL) and 10 (1 × 10^6^ CFU/mL) for 2 h. Following incubation, monolayers were washed five times with 1 × PBS to remove non-adherent bacteria and lysed with 0.1% Triton X-100 (Millipore Sigma, MA, USA) for 3 min at 125 rpm to release cell-associated bacteria (i.e., adherent and internalized bacteria). Lysates were serially diluted and plated for CFU enumeration.

For the invasion assay, the supernatants and extracellular bacteria were gently aspirated after 2 h post-infection. The cocultured cells in each well were washed and incubated for another 2 and 4 h in culture medium containing 100 µg/mL gentamicin and 5 µg/mL penicillin G to kill extracellular *S. suis* (internalized bacteria are spared). The cell monolayers were then washed 3 times with sterile 1xPBS and lysed with 0.1% Triton X-100 (MERCK, Millipore, MA, USA) in 1 × PBS for 3 min while shaking at 125 rpm to release intracellular bacteria.

The adhesion and invasion of bacteria were quantified on BHI agar plates as CFUs after 24 h incubation at 37 °C as previously described. Results were reported as the percentage of internalized bacteria compared to the inoculated bacteria from 3 biological replicates. The percentage of bacterial adhesion and internalization was calculated as the relative number of adhered and internalized bacteria to the inoculum. Assays were performed in triplicate and repeated at least three times.

### Mouse model of *S. suis* infection

Specific pathogen-free (SPF) 7-week-old female A/J mice were purchased from Japan SLC (Shizuoka, Japan). Mice (10 mice per group) were inoculated intraperitoneally. For the determination of dose-dependent lethality, the WT strain was injected into mice with different doses, including 1 × 10^9^, 5 × 10^8^, 1 × 10^8^, and 1 × 10^7^ colony-forming units (CFUs). To compare the survival rate of WT, *Δsbp1*, and *Δsbp3* strains, mice were inoculated with 1 × 10^8^ CFU of WT and mutants. Survival was monitored for 14 days.

The dose-dependent lethality experiment and the WT-versus-mutant survival experiment were performed independently using separate cohorts of mice. For the mutant virulence experiment, a WT control group was included and tested concurrently with the mutant strains.

### Statistical analysis

Data were expressed as mean ± standard deviation (SD) values. Comparisons between WT, *Δsbp1,* and *Δsbp3* mutant strains were investigated using one-way ANOVA (Post Hoc Multiple Comparisons; Tukey). The log-rank test was used to compare the survival rates between the *S. suis*-infected mouse strains. The statistical significance of the differences between the groups was indicated as *p < 0.05, **p < 0.01, or ***p < 0.001.

### Ethics

Research protocols were reviewed and approved by the Human Research Ethics Subcommittee, Rajamangala University of Technology Isan Sakon Nakhon Campus, Thailand (Certificate exemption: HEC-04–68-010). Mouse experiments were approved by the Ethics Committee of The University of Osaka (Certificate number H21-09-0).

## Results

### Growth of *S. suis sbp*-knockout strains

**Figure 1:**
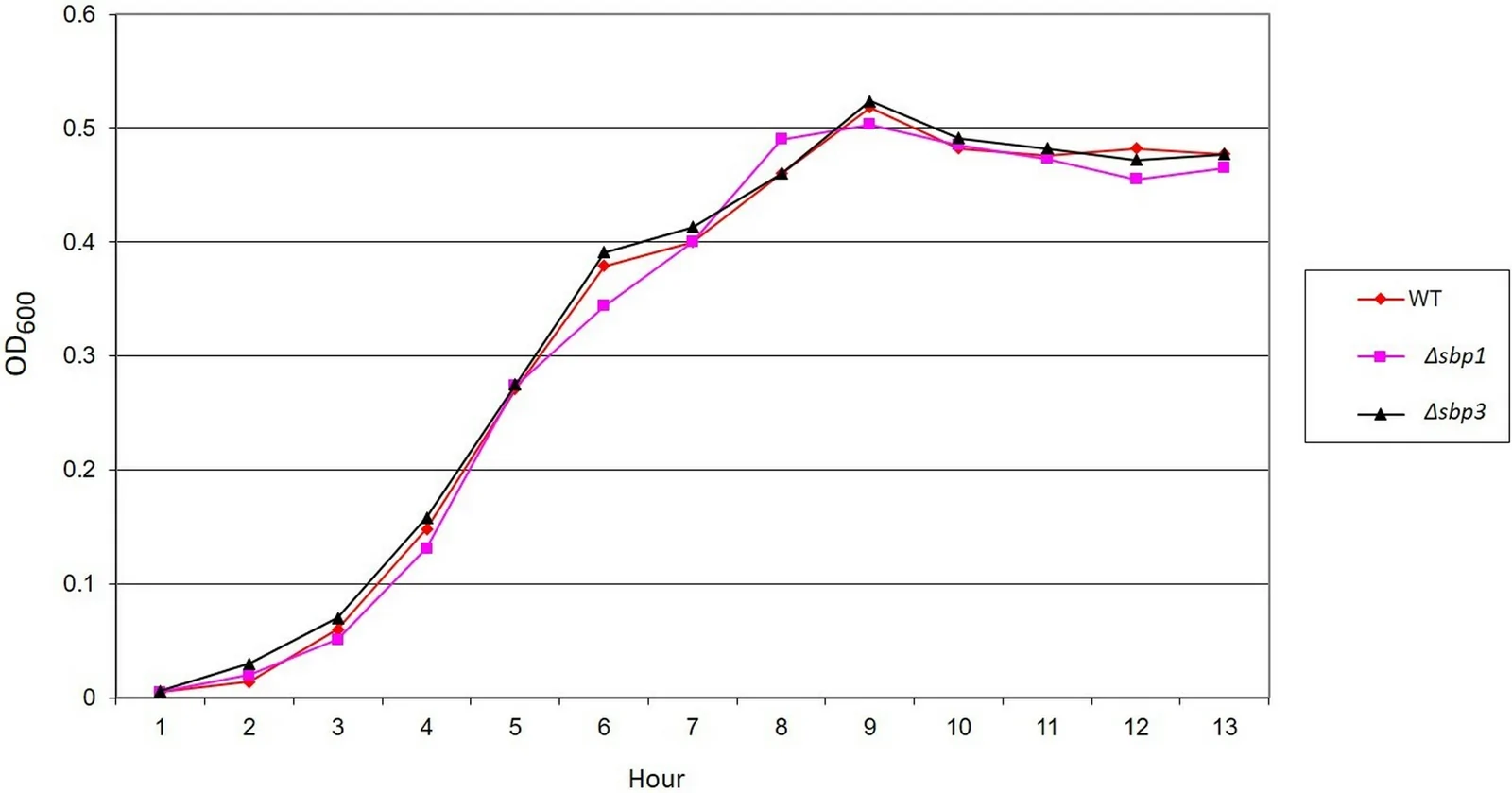
***Streptococcus suis* serotype 2 ST1 strain 26154 and its *Δsbp1* and *Δsbp3* mutants in Todd–Hewitt broth**. Optical density at 600 nm (OD_600_) was measured at the indicated time points.

Allelic replacement mutants of *sbp1* and *sbp3* were generated in the *S. suis* serotype 2 ST1 strain 26154 by insertion of a chloramphenicol resistance cassette (*cat*). Correct integration was verified by PCR using primers flanking the recombination sites and by DNA sequencing. The resulting mutants were designated *Δsbp1* and *Δsbp3*. To assess whether disruption of *sbp1* or *sbp3* affected bacterial fitness, growth kinetics of the WT and mutant strains were evaluated in Todd–Hewitt broth. Both mutants had growth curves comparable to the WT strain, indicating that inactivation of these genes did not alter in vitro growth (Figure 1).

### Biofilm formation of *S. suis sbp*-knockout strains

**Figure 2:**
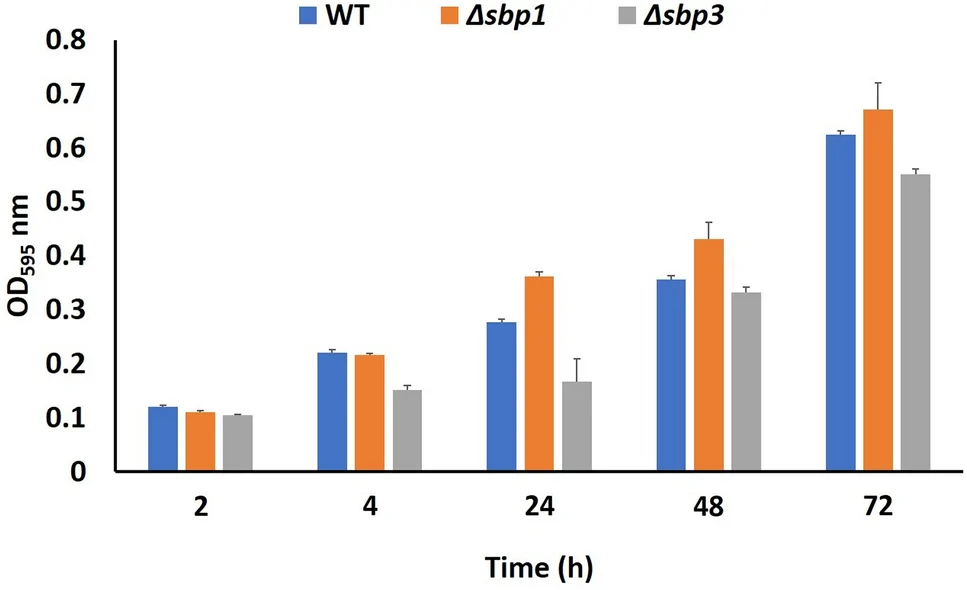
**Biofilm formation of *S. suis* WT, *Δsbp1* and *Δsbp3* strains**. The crystal violet staining of biofilms formed in 96-well microplates and quantified at an absorbance of 595 nm at different time points. Data represent three independent experiments. Statistical significance compared with WT (*p < 0.05, **p < 0.01, ***p < 0.001)**.**

Biofilm formation of the *S. suis* WT, *Δsbp1* and *Δsbp3* strains was evaluated at 2, 4, 24, 48, and 72 h. At 2 h (Figure 2), the *Δsbp1* (*p* < 0.05) and *Δsbp3* (*p* < 0.01) strain exhibited significantly decreased biofilm formation as compared to WT strain. This attenuation was also evident at 4 h, where only the *Δsbp3* strain continued to produce significantly lower biofilm biomass than the WT (*p* < 0.001). At 24, 48, and 72 h, the difference became even more pronounced, with the *Δsbp3* strain showing significantly diminished biofilm development relative to the WT, indicating an impairment in early biofilm establishment. However, the biofilm formation of the *Δsbp1* strain significantly increased after 24, 48, and 72 h of incubation when compared to the WT strain (Figure 2).

### Transcriptional analysis of pilus cluster and virulence-associated genes in *S. suis sbp*-knockout strains

**Figure 3:**
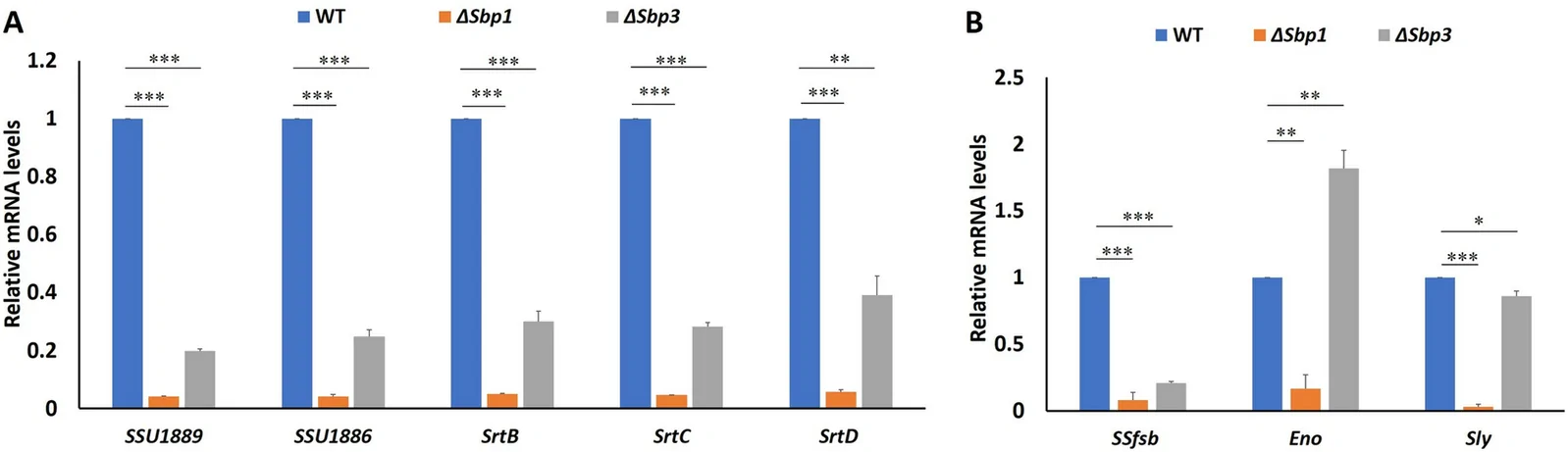
**Relative expression of adhesion- and cytotoxic-associated genes by the *S. suis* WT, *Δsbp1*, and *Δsbp3* strains**. The mRNA level of **A** neighboring genes in *srtBCD* cluster including *SSU1889, SSU1886, SrtB, SrtC,* and *SrtD*, **B** adhesin genes (*ssfsb, eno*), and suilysin gene *(sly*).

The relative expression levels of *SSU1889, SSU1886, srtB, srtC,* and *srtD* within the *srtBCD* cluster were significantly reduced in both the *Δsbp1* and *Δsbp3* mutants compared with the WT strain, as determined by RT-qPCR analysis (Figure 3A). However, the reduction was markedly greater in the *Δsbp1* mutant, in which transcript levels were nearly abolished relative to the WT. In contrast, the *Δsbp3* mutant retained substantially higher expression levels of these genes than the *Δsbp1* mutant.

The relative expression of *ssfsb*, *eno*, and *sly* was further examined by qRT-PCR (Figure 3B). Expression of *ssfsb* was significantly reduced in both the *Δsbp1* and *Δsbp3* mutants compared with the WT strain, with the lowest transcript level observed in the *Δsbp1* mutant. In contrast, expression of *eno* was significantly increased in the *Δsbp3* mutant (approximately 1.8-fold relative to the WT), whereas it remained significantly reduced in the *Δsbp1* mutant. Expression of *sly* was also significantly decreased in both mutants, with the most pronounced reduction detected in the *Δsbp1* mutant.

### Cytotoxicity of *S. suis sbp*-knockout strains to human epithelial cells

**Figure 4:**
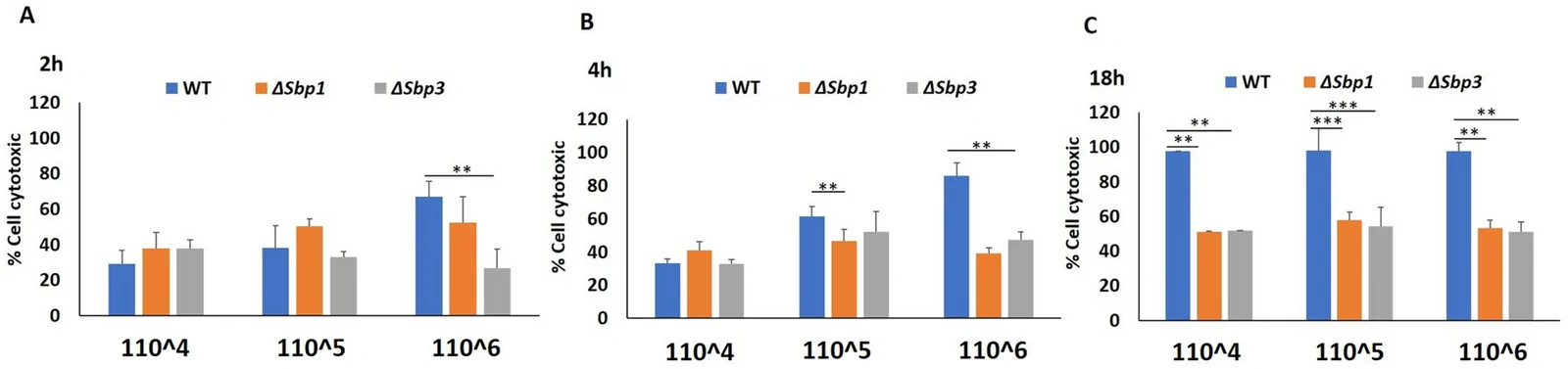
**Cytotoxicity of the wild-type and mutant derivative *Δsbp1* and *Δsbp3* strains towards A549 cells**. Percentage of cytotoxicity cells after being infected with three strains at MOI 1, MOI 10, and MOI 100 at (**A**) 2 h, (**B**) 4 h, and (**C**) 18 h. Data represent means ± SD from three independent experiments performed in triplicate. Statistical significance compared with WT is indicated by asterisks (*p < 0.05, ** *p* < 0.01, ***p < 0.001).

To evaluate cytotoxicity, A549 cells were infected separately with WT, *Δsbp1*, or *Δsbp3* strains at different MOIs. The cytotoxicity of the WT strain was significantly greater than that of the *Δsbp3* mutant strains at an MOI of 100, 2 h post-infection (*P* < 0.01; Figure 4A). Figure 4B shows that the WT strain induced significantly more cytotoxicity than *Δsbp1* (*P* < 0.01) or *Δsbp3* (*P* < 0.01) mutant strains at MOI of 100 at 4 h post-infection (Figure 4B). At 18 h post-infection, *Δsbp1* and *Δsbp3* induced significantly decreased epithelial cell cytotoxicity at MOI1 (*P* < 0.01), MOI10 (*P* < 0.001), and MOI100 (*P* < 0.01) when compared to the WT strain (Figure 4C). Collectively, these results indicate that disruption of *sbp1* or *sbp3* attenuates *S. suis*-induced epithelial cell cytotoxicity.

### Adhesion and invasion activity of the *sbp*-knockout strains to human epithelial cells

To assess the role(s) of *sbp* genes, we next investigated the capacity of the three strains to adhere to and invade A549 cells. The percentage of adherent bacteria associated with A549 cells following infection with the *Δsbp1* and *Δsbp3* mutants at MOI 1 was 0.03 ± 0.01% and 0.02 ± 0.01%, respectively. At MOI 10, adhesion increased to 0.94 ± 0.35% for *Δsbp1*, whereas *Δsbp3* remained low at 0.02 ± 0.002% (Table 2 and Additional file 2). The results of the bacterial cell adhesion showed that the number of viable bacteria on the surface and internalization of A549 cells of *Δsbp1* (*P* < 0.05) and *Δsbp3* (*P* < 0.01) mutant strains was significantly reduced compared to WT at MOI 1 (Table 2 and Additional file 2). However, the adherent cells were significantly increased after infection with *Δsbp1* (*P* < 0.01) compared to the WT strain (Table 2 and Additional file 2).

**Table 2:** **Percentage adhesion of wild-type and mutant** ***Streptococcus suis*** **strains to A549 epithelial cells at different multiplicities of infection (MOIs)**

% cell adhesion	Wild-type strain	*Δsbp1* mutant	*Δsbp3* mutant
MOI 1	0.089 ± 0.025	0.03 ± 0.01	0.02 ± 0.01
MOI 10	0.002 ± 0.001	0.94 ± 0.35	0.02 ± 0.002

Data are presented as mean ± standard deviation from three independent experiments

Invasion assays showed no detectable intracellular bacteria for any strain at MOI 1 after infected with 2 h (Table 3 and Additional file 2) and 4 h (Table 3 and Additional file 2). At MOI 10 after 2 h of infection, the WT strain produced low but measurable invasion (0.015 ± 0.002%), whereas no intracellular bacteria were detected for either the *Δsbp1* or *Δsbp3* mutants. Accordingly, invasion by both mutants was significantly reduced compared to the WT at MOI 10 (*P* < 0.001 for both comparisons) as shown in Table 3 and Additional file 2. At MOI 10 after 4 h of infection, the invasion of WT (0.012 ± 0.004%) was significant higher the *Δsbp1* (0.007 ± 0.004%), whereas no significant in *Δsbp3* (0.012 ± 0.003%) mutants (Table 3 and Additional file 2).

**Table 3:** **Percentage invasion of wild-type and mutant** ***Streptococcus suis*** **strains into A549 epithelial cells at different multiplicities of infection (MOIs) and incubation times**

Time (h)	% cell invasion	Wild-type strain	*Δsbp1* mutant	*Δsbp3* mutant
2	MOI 1	0	0	0
	MOI 10	0.015 ± 0.002	0	0
4	MOI 1	0.006 ± 0.010	0	0
	MOI 10	0.012 ± 0.004	0.007 ± 0.004	0.012 ± 0.003

Data are presented as mean ± standard deviation from three independent experiments.

### Mouse survival

**Figure 5:**
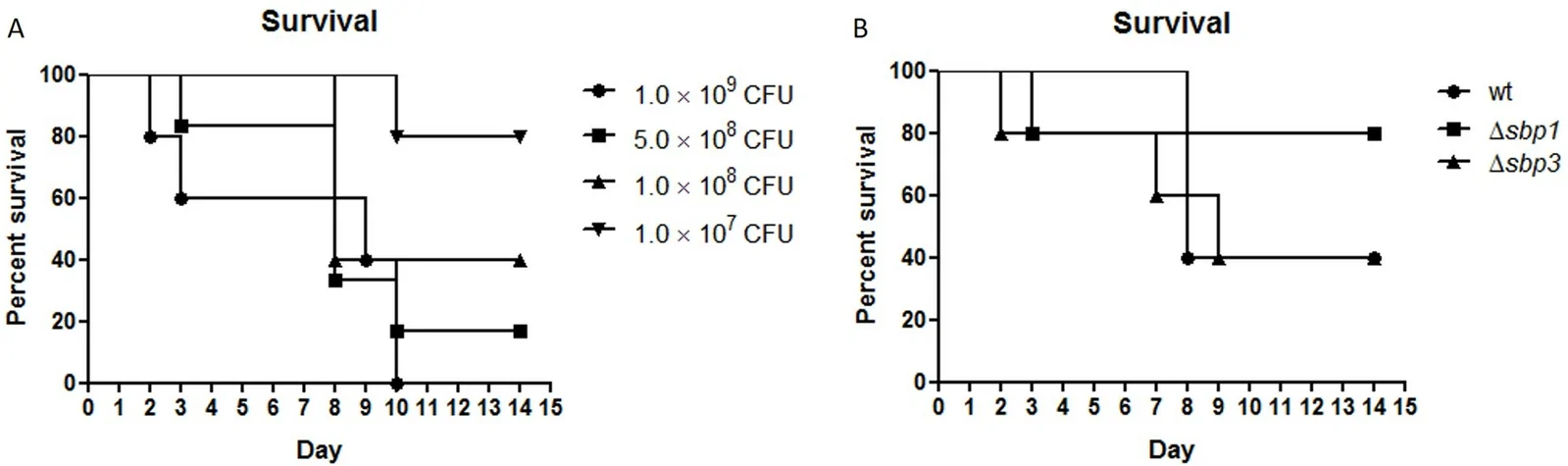
**Mouse survival induced by *S. suis* serotype 2-ST1 strain 26154 of wild type, *Δsbp1* or *Δsbp3* mutants**. **A** Mouse survival by different doses of a wild-type strain. **B** Mouse survival by wild type, *Δsbp1* and *Δsbp3* at 1 × 10^8^ CFU injection dose. Statistical analysis was determined by the log-rank test (*p* = 0.463).

As shown in Figure 5A, WT (strain 26154) caused dose-dependent mortality in mice. At 1 × 10^9^ CFU, mortality began on day 2, and all mice died by day 10. At 5 × 10^8^ CFU, mortality started on day 3, with 20% survival at day 14. At 1 × 10^8^ CFU, mortality was delayed until day 8, and 40% of mice survived to day 14. In contrast, infection with 1 × 10^7^ CFU resulted in 80% survival at day 14.

To compare mutant virulence, mice were inoculated with 1 × 10^8^ CFU of WT, *Δsbp1*, or *Δsbp3* (Figure 5B). Infection with *Δsbp1* resulted in 80% survival at day 14, which is higher than that of WT. In contrast, *Δsbp3* produced a survival rate (40%) comparable to WT. However, the log-rank test showed no statistically significant differences in mouse survival rates among the WT, *Δsbp1*, and *Δsbp3* groups (*p* = 0.463). These findings indicate that *sbp1* seems to play a greater role than *sbp3* in the pathogenicity of *S. suis*, however, neither gene inactivation had a significant effect on mouse mortality under the conditions tested.

## Discussion

The *srtBCD* pilus cluster is associated with virulent *S. suis* isolates, and appears to be largely restricted to CC1 isolates [7, 8]. The cluster encodes four pilin subunit proteins, *sbp1-sbp4*, located upstream of three type C sortases (*srtB, srtC*, and *srtD*) [7]. Several studies have demonstrated the involvement of this locus in virulence [11–13, 19]. For example, deletion of *srtB* of *S. suis* strain P1/7 (ST1) attenuated the virulence in caesarean-derived colostrum-deprived pigs [20]. The *sbp2’* of *S. suis* serotype 2 ST7 (strain ZY05719) contributed to adhesion and virulence as assessed with a zebrafish larvae model of infection [12]. A study by Qian et al. demonstrated that *sbp1* in the highly virulent *S. suis* strain ZY05719 (ST7) primarily functions as an adhesion-associated factor, contributing to the initial attachment of bacteria to host cells rather than to subsequent pathogenic processes such as invasion, intracellular survival, or virulence [13].

In this study, using an ST1 serotype 2 Thai strain, our findings suggest that *sbp3* may contribute to early pathogen–host interactions. However, no significant difference in adherence was observed, and the overall invasion efficiency of the strain was low, limiting conclusions regarding the role of *sbp3* in bacterial adherence and invasion. These findings differ somewhat from those of Sunontarat et al., who identified *sbp3* as a virulence-associated gene involved in adhesion, invasion, and cytotoxicity in human intestinal epithelial cells [16]. The discrepancies between the two studies may be partly explained by differences in the epithelial cell models used (Caco-2 intestinal epithelial cells versus A549 lung epithelial cells), which possess distinct receptor repertoires and cellular responses that can influence bacterial adhesion and internalization. Consequently, the contribution of *sbp3* to host-cell interactions may be dependent on the host-cell type and experimental context.

In contrast, *sbp1* appeared to play a more prominent role in invasion than *sbp3*, as the *Δsbp1* mutant exhibited significantly reduced invasion at MOI 10. Moreover, an unexpected increase in adhesion was observed for the *Δsbp1* mutant at the same MOI. These findings differ from those reported by Qian et al. [13], who showed that deletion of *sbp1* in the ST7 strain ZY05719 significantly reduced adherence to HEp-2 epithelial cells but had no effect on bacterial invasion. Several factors may account for these discrepancies. First, different epithelial cell models were used in the two studies (A549 lung epithelial cells versus HEp-2 epithelial cells), which express distinct surface receptors and may influence bacterial adhesion and internalization mechanisms. Second, the strains belong to different genetic backgrounds (ST1 versus ST7), potentially possessing distinct regulatory networks and accessory gene repertoires that affect pilus-associated functions. Finally, methodological differences, including infection dose and incubation period, may have influenced the detection of low-frequency invasion events. Collectively, these findings suggest that although the role of SBP1 in host-cell adhesion may be conserved across *S. suis* lineages, its contribution to invasion is likely strain- and host-cell-dependent, underscoring the complex functions of pilus-associated proteins in *S. suis* pathogenesis.

Interestingly, adhesion by the *Δsbp1* mutant was significantly increased at higher MOI, suggesting that *sbp1* may not function solely as a classical adhesin. Consistent with this observation, the *Δsbp1* mutant did not impair biofilm formation and instead resulted in increased biofilm biomass, particularly during the maturation phase (24–72 h). The enhanced adhesion phenotype may therefore contribute to the increased biofilm accumulation observed in the mutant. However, the mechanisms underlying these phenotypes remain unclear. It is possible that loss of *sbp1* alters bacterial surface properties or affects the expression and/or surface exposure of other adhesins, thereby promoting attachment and subsequent biofilm development. Similar compensatory effects have been reported in other Gram-positive bacteria. For example, deletion of pilus-associated adhesins in *Streptococcus gordonii* has been associated with increased adhesion or biofilm formation through altered expression of other adhesin genes and the exposure of alternative adhesion mechanisms [21]. Further studies are required to determine whether similar regulatory or surface-associated changes occur in the *Δsbp1* mutant.

Mutation of *sbp1* and *sbp3* may interfere the cytotoxicity-associated gene expression, such as suilysin or other hemolysins. This dysregulation could lead to changes in host cell damage, impair bacterial virulence, and affect the overall interaction between the pathogen and host immune responses. Our results suggest that the reduced cytotoxicity observed in the *Δsbp1* and *Δsbp3* mutants may be mediated, at least in part, by decreased expression of *sly*, as both mutants exhibited significantly lower *sly* transcription levels than the wild-type strain in the present study. Consistent with this finding, a previous study has demonstrated that deletion of *sbp3* results in reduced *sly* expression, supporting a regulatory link between pilus-associated components and suilysin production [16].

RT-qPCR analysis revealed that inactivation of *sbp1* and *sbp3* significantly affected transcription of genes within the *srtBCD* pilus island and several virulence-associated genes. The effect was most pronounced in the *Δsbp1* mutant, which exhibited markedly reduced expression of *SSU1889*, *SSU1886*, *srtB*, *srtC*, and *srtD*, suggesting that *sbp1* is important for maintaining transcription of the pilus locus. Both mutants also showed reduced expression of *ssfsb* and *sly*, indicating that disruption of pilus-associated components influences additional virulence determinants. In contrast, *eno* expression was significantly upregulated in the *Δsbp3* mutant, suggesting a potential compensatory response through increased production of alternative surface-associated adhesins. These findings may support a role for *sbp1* and *sbp3* beyond pilus structure, potentially contributing to the coordinated regulation of virulence-associated genes in *S. suis*.

In vivo, no statistically significant differences in survival were detected among the WT, *Δsbp1*, and *Δsbp3* groups, suggesting that neither *sbp1* nor *sbp3* plays a major role in mouse mortality. These findings are consistent with a previous study demonstrating that *sbp1* inactivation in an ST7 background did not significantly affect *S. suis* virulence in zebrafish or mouse infection models [13]. Nevertheless, the *Δsbp1* mutant showed a trend toward greater mouse survival than the WT and the *Δsbp3* mutant. The precise mechanism by which *sbp1* contributes to virulence remains unclear. Although inactivation of *sbp1* resulted in attenuated virulence in the mouse model, the current findings do not support a simple interpretation that *sbp1* functions solely as a classical surface adhesin directly mediating systemic infection. Recent evidence highlights the complexity of the *srtBCD* system in *S. suis*. Although no classical pilus structures were detected, the truncated major pilin subunit SBP2′ was found on the bacterial surface and contributed to virulence through plasminogen binding, whereas SBP1, SBP3, and SBP4 were not detectably surface-localized under the tested conditions [19]. These findings suggest that *srtBCD*-associated proteins may have distinct biological functions and may perform distinct functions in host–pathogen interactions independent of classical pilus formation.

Instead, our results hypothesize that *sbp1* may contribute to virulence through indirect or context-dependent mechanisms. One possibility is that *sbp1* is involved in the processing, assembly, or functional regulation of cell-envelope-associated structures, thereby influencing the expression, surface accessibility, or activity of other virulence-associated factors. This hypothesis is supported by the observed transcriptional changes following *sbp1* deletion, including altered mRNA expression of *sly*, *eno*, *ssfsb*, and neighboring genes within the *srtBCD* cluster. Alternatively, under specific environmental or host conditions, *sbp1* may be released or differentially presented, enabling interactions with bacterial or host components that contribute to pathogenesis without requiring constitutive surface exposure. These proposed mechanisms remain speculative and are not directly demonstrated by the present study. Therefore, further investigations are needed to determine the localization and potential release of *sbp1*, characterize its interactions with other virulence-associated proteins, and evaluate the transcriptional and proteomic consequences of *sbp1* deletion. Such studies will be essential for clarifying the contribution of *sbp1* to *S. suis* pathogenesis.

Collectively, our findings support a role for the *srtBCD* pilus cluster as a virulence-associated locus in *S. suis*. Together with previous studies implicating *srtB*, *sbp1*, *sbp2’*, and *sbp3* [12, 13, 16, 19, 20], the present findings further emphasize the pilus-associated components *sbp1* and *sbp3* in mediating early pathogen–host interactions. In conclusion, inactivation of *sbp1* and *sbp3* impaired the ability of *S. suis* to damage epithelial cells, adhere to host cells, and invade them. Deletion of either gene also reduced the expression of the sortase-associated gene cluster (*SSU1889, SSU1886, srtB, srtC, srtD*), and several virulence-associated genes, including the adhesin genes *ssfsb*, *eno*, and *sly*, indicating that *sbp1* and *sbp3* may contribute to a broader virulence regulatory network. Among the two proteins, *sbp1* appeared to exert a greater influence on virulence, as the *Δsbp1* mutant exhibited more pronounced reductions in virulence gene expression. Although neither *sbp1* nor *sbp3* significantly affected mouse mortality, the *Δsbp1* mutant showed a trend toward increased mouse survival compared with the *Δsbp3* mutant.

Some limitations should be acknowledged. First, A549 lung epithelial cells were used as the sole in vitro model. Although respiratory manifestations of *S. suis* infection have been reported in humans and pigs, A549 cells do not fully represent the primary portals of human infection, namely the gastrointestinal tract and damaged skin. Therefore, the observed host–pathogen interactions may not fully reflect those occurring during natural infection. Second, the in vivo virulence experiments were performed exclusively in A/J mice. Although A/J mice are considered susceptible to *S. suis* serotype 2 infection, the WT strain used in this study exhibited relatively attenuated virulence compared with virulent reference strains, potentially reducing the sensitivity of the model to detect virulence differences associated with *sbp1* or *sbp3* inactivation. Consequently, the magnitude and generalizability of the observed phenotypes should be interpreted with caution. Future studies using additional epithelial cell models and complementary animal models are needed to further validate the roles of *sbp1* and *sbp3* in *S. suis* pathogenesis.

## Supplementary Information

**Additional file 1. Schematic representation of the construction of *Δsbp1*** **and** ***Δsbp3*** **mutants in** ***S. suis*** **strain 26154. **Upstream and downstream fragments of *sbp1* or *sbp3* were amplified and fused to a chloramphenicol resistance cassette (cat) for allelic replacement. Recombinant plasmids (pSBP1 and pSBP3) were used to generate gene disruption mutants in strain 26154. Correct integration was confirmed by PCR using diagnostic primers.

**Additional file 2. Adhesion to and invasion of A549 cells by the wild-type and mutant derivative *Δsbp1*** **and *Δsbp3*** **strains. **(A) Percentage of cell-associated (adherent) bacteria. (B) Percentage of intracellular (invaded) bacteria at 2 h post-gentamicin protection assay. (C) Percentage of intracellular (invaded) bacteria at 4 h post-gentamicin protection assay. For each strain, adhesion and invasion assays were performed in triplicate. Data represent means ± SD from three independent experiments performed in triplicate. Statistical significance compared with WT is indicated by asterisks (*p < 0.05, **p < 0.01, ***p < 0.001).

## Data Availability

The datasets used and/or analyzed during the current study are available from the corresponding author on reasonable request.
